# The Relationship Between Motor Development and ADHD: A Critical Review and Future Directions

**DOI:** 10.3390/bs15050576

**Published:** 2025-04-24

**Authors:** Gabrielle A. Shimko, Karin H. James

**Affiliations:** Department of Psychological and Brain Sciences, Indiana University, Bloomington, IN 47405, USA; khjames@iu.edu

**Keywords:** ADHD, motor development, embodied cognition

## Abstract

Despite the prevalence of motor difficulties in individuals with attention-deficit/hyperactivity disorder (ADHD) throughout development, it is neglected as a factor underlying the etiology or functional impairment. This paper reviews the behavioral and neurological evidence linking motor difficulties to ADHD, arguing that existing theories explaining this relationship are oversimplified. Instead, embodied theories of development offer a more comprehensive evaluation of the mechanistic relationship among the motor system, cognitive development, and subsequent functional impairment in ADHD throughout early development. The integration of these perspectives will ultimately inform our understanding of the etiology of ADHD and inspire novel approaches for identification and intervention.

## 1. Introduction

Developmental psychology has long recognized that early motor behavior is foundational to perceptual, social, and cognitive development. The acquisition of basic motor skills, such as sitting upright, crawling, or gaining control over limbs and hands to hold and interact with objects, can be understood as part of an interactive developmental process that enables new opportunities for exploratory interactions with a complex environment (Adolph & Hoch, 2019; Smith & Sheya, 2010; Thelen, 2000). Repeated, adaptive, and flexible motor behaviors and perceptual experiences subsequently give rise to new motor skills and thus facilitate learning, cognitive, and social development. Beyond motor development’s central, interactive role in supporting cognitive development, the acquisition of complex manual skills, such as handwriting, critically supports academic achievement (Cameron et al., 2016; James, 2017).

Given the inseparable nature of cognitive and motor development, delays or impairments in motor skills can have cascading effects on cognitive and behavioral outcomes. Attention-deficit/hyperactivity disorder (ADHD) is a prevalent neurodevelopmental disorder characterized by developmentally inappropriate levels of inattentiveness and hyperactivity/impulsivity, leading to significant impairments in school, home, and occupational settings (American Psychiatric Association, 2013). Although not traditionally viewed as part of the diagnostic criteria, motor difficulties are frequently observed in children with ADHD, including deficits in fine and gross motor skills (Goulardins et al., 2017; Kaiser et al., 2015). Notably, it is estimated that up to half of children with ADHD meet diagnostic criteria for developmental coordination disorder (DCD), a neurodevelopmental disorder defined by persistent difficulties in learning and executing coordinated movements despite adequate opportunities for development (American Psychiatric Association, 2013; Meachon et al., 2025; Pranjić et al., 2023; Wilson et al., 2020). Even in the absence of formal DCD diagnoses, children with ADHD consistently demonstrate a range of motor difficulties relative to neurotypical peers, though often less severe than those with comorbid DCD (Goulardins et al., 2013, 2017, 2024; Lee et al., 2021; Lin et al., 2024; McLeod et al., 2014). Importantly, longitudinal evidence suggests that early motor difficulties in the context of ADHD symptoms predict poorer occupational outcomes in adulthood, underscoring the long-term functional impact of these challenges (Landgren et al., 2022).

Despite evidence of persistent motor difficulties in ADHD from infancy (Athanasiadou et al., 2020; Reetzke et al., 2022) into adulthood (Fietsam et al., 2022; McCracken et al., 2023) and the high rate of comorbidity with DCD, motor difficulties are rarely considered as an important feature related to the etiology or functional impairment associated with ADHD symptoms. This is in contrast to years of accumulated research in developmental psychology that suggest motor and cognitive development are inseparable (Adolph & Hoch, 2019; Thelen, 2000). Therefore, there is a need to bridge these detached lines of research between clinical and developmental science to contextualize and make meaning of consistent indications of motor difficulties in ADHD.

The purpose of this review is to examine the relationship between motor development and ADHD and to argue that drawing upon embodied theories of development is crucial for understanding the potential neurodevelopmental role of the motor system in ADHD. First, embodied theoretical frameworks will be briefly summarized to inspire a more nuanced perspective on the relationship between motor development and functional difficulties associated with ADHD symptoms throughout development. We will characterize behavioral and neurological evidence relating motor development and skills to ADHD during infancy and throughout childhood and argue that the prevalent theories positing why children with ADHD exhibit motor difficulties are insufficient. Ultimately, we propose that the adoption of an embodied perspective will advance our understanding of the etiology of ADHD, which has important implications in early identification and intervention.

## 2. Motor Development as an Embodied Process

Piaget (1952) was perhaps the first developmentalist to consider how actions and activity patterns during infancy were foundational for cognitive development. Built on this, embodied theories of development center motor activity and development within behavioral development. From this perspective, increasing mobility and action control affords new opportunities for learning that support development in a wide range of psychological domains typically deemed as disparate (Adolph et al., 1997; Smith & Sheya, 2010; Smith & Thelen, 2003; Thelen, 2000). That is, motor behaviors or actions are not secondary to internal cognitive processes, but rather, actions performed within an environment shape cognitive systems by adapting to our environment (Shapiro, 2019). For example, infants are not merely learning to crawl because the brain passively reaches a maturational milestone, but rather, crawling emerges as a functionally significant, goal-directed behavior that enables environmental exploration and novel, multimodal experiences (Adolph & Kretch, 2015; Bertenthal & Campos, 1990; Gibson, 1988).

Developing action control, such as learning to independently sit upright or control limb movements, enables infants and children to explore and sample their environment in increasingly complex ways. Specifically, acting to obtain information, such as by moving their eyes or head to scan a scene or by grasping or mouthing an object to learn its properties, supports perceptual development by enabling infants to actively acquire information (Adolph & Kretch, 2015; Gibson, 1988). New motor skills or actions offer new opportunities for action in the environment and require the child to generate novel behavioral solutions, allowing for the child to continuously uncover possibilities for action within a dynamic environment through a process of matching an individual’s physical and perceptual abilities to current environmental demands (Adolph & Hoch, 2019; Gibson, 1988).

Motor development and sensorimotor engagement with the environment and objects affect brain changes, facilitating the groundwork for future cognitive and perceptual abilities in typical and atypical development (Belsky & de Haan, 2011; Byrge et al., 2014; M. H. Johnson & de Haan, 2015). Executive function, or the ability to choose, enact, and sustain goal-directed behaviors via inhibition, working memory, and flexibility, is an important predictor for outcomes such as ADHD, and difficulties in executive function are proposed to underlie the presentation of ADHD symptoms (Barkley, 2011; Gottwald et al., 2016). Executive function is often discussed as being localized in the frontoparietal network and as developing in a hierarchal manner, which would subsequently affect motor skills in a top-down fashion (Gottwald et al., 2016; L. F. Koziol & Lutz, 2013). However, the frontoparietal network is itself embedded within distributed, earlier-developing sensorimotor systems (Casey et al., 2005; Cisek & Kalaska, 2010; L. F. Koziol & Lutz, 2013). The neural mechanisms for action control scaffold executive function development, such as the ability to control and plan actions, allow children to repeatedly explore dynamic, novel environments and adaptively anticipate sensorimotor outcomes (Adolph & Kretch, 2015; Cisek & Kalaska, 2010; Gottwald et al., 2016; L. F. Koziol & Lutz, 2013). Indeed, the capacity for complex action planning emerges during infancy, and thus, prior to measurable executive function, which continues to proliferate into early childhood (Thelen et al., 1996; Von Hofsten, 2004, 2009). Thus, early action control may serve as a developmental precursor to executive function.

Action control eventually generates semantic knowledge that proactively biases future interactions, decision making, and behavioral flexibility—which underpins the foundation of later executive function that emerges in toddlerhood (Gottwald et al., 2016; Sheya & Smith, 2010). Within this process, how information is extracted, selected, and optimized through action largely depends on the level of experience/previous learning, and an individual’s motor and perceptual capabilities enable, or constrain, applying new strategies to novel challenges in an environment (Adolph & Kretch, 2015; Gibson, 1988; Thelen et al., 1993). Enriched and responsive caregiving environments during early development seem to help scaffold exploration of the environment, which subsequently affects later pre-frontal cortex and executive function development into childhood (Rosen et al., 2019). Thus, the development of action control during infancy, such as reaching or walking, and utilizing these actions to support exploratory goals, subserves typical cognitive development. Importantly, difficulty or differences in these actions should be evaluated in understanding the emergence of ADHD phenotypes later in development, given the relationship between action control and executive function.

Increased autonomy via action control during the first two years of life is critical in developing cognitive systems, such as sustained attention and executive function. The quantity and variety of object exploration via manual control drives learning about objects and subsequently drives cognitive development (Mendez et al., 2024; Soska & Adolph, 2014; Yu et al., 2019). Through postural control, the child’s hands are now free for visual-guided reaching, manipulation, and grasping of objects to bring them closer to their face or visual field for bimanual, multimodal (mouthing and viewing) exploration (Soska & Adolph, 2014; Yang et al., 2023). Head and postural stability enable infants to reduce visual input variability and maintain the visual salience of an object of interest over distractors by bringing it close to the head for a period of several seconds, or through sustained attention (Mendez et al., 2024). Thus, increased postural and manual control facilitates multimodal, sustained visual attention to objects. Multimodal object attention subsequently facilitates better object recognition, more object exploration, vocabulary growth via joint attention, and importantly, sustained attention and inhibitory control later in development (Kretch et al., 2022; Libertus et al., 2016; Mendez et al., 2024; Needham et al., 2002; Rosen et al., 2019; Schroer & Yu, 2023; Soska & Adolph, 2014; Soska et al., 2010; Wijnroks & Veldhoven, 2003; Yang et al., 2023; Yu et al., 2019; Yuan et al., 2019).

Additionally, independent mobility, or learning to crawl or walk, enables infants to actively explore new people, places, and objects within their environment, further supporting the development of cognitive systems (Anderson et al., 2013; Campos et al., 2000; Karasik et al., 2011). Children who were early crawlers exhibited better spatial-cognitive memory task performance, such as better mental rotation abilities, performance on spatial search tasks, and more flexible action applications, which may be due to the increased array of object views locomotion offers (Adolph & Hoch, 2019; Bertenthal & Campos, 1990; Campos et al., 2000; Clearfield, 2004; Schwarzer et al., 2013). Walkers carry objects more frequently, which facilitates increased manual exploration and joint attention with caregivers, which may have cascading effects on attentional and vocabulary development (Clearfield, 2011; Karasik et al., 2011; Walle, 2016; Yang et al., 2023). Even artificial mobility, such as allowing pre-walking infants to use a walker, seems to accelerate cognitive milestones, such as earlier success in the A-not-B error task (Kermoian & Campos, 1988).

These observations suggest that increased autonomy via action control during the first two years of life is critical in developing cognitive systems, such as sustained attention and executive function, and thus, could potentially be early markers for neurodevelopmental disorders like ADHD (Berger et al., 2019). Difficulties with mobility or action control during key developmental windows, particularly in infancy, may contribute to cascading challenges across motor, cognitive, and academic domains that characterize the ADHD phenotype (Pant et al., 2022). For example, lower sustained attention on objects during play in infancy predicts worse sustained attention and hyperactivity problems by preschool, particularly in reactive temperaments common in ADHD (M. H. Johnson et al., 2015; Lawson & Ruff, 2004a). There is evidence that delays in postural stability, crawling, and walking during infancy may be related to ADHD in childhood, highlighting potential mechanisms by which difficulties with action control may subserve atypical cognitive development (Gurevitz et al., 2014; Lemcke et al., 2016; Pant et al., 2022). For example, reduced bimanual object exploration near the face, during a sensitive period for developing visual sustained attention, may limit opportunities for object manipulation and joint attention with caregivers, in turn weakening the development of sustained attention skills and contributing to behavioral and academic difficulties later associated with ADHD (Kofler et al., 2019; Kretch et al., 2022; Preston et al., 2009; Tucha et al., 2009; West & Iverson, 2017; Yuan et al., 2019).

Beyond action control’s role in cognitive development, early manual skills and action control lay the foundation for future motor skills into childhood. Early manual skills shape the development of sensorimotor integration, coordination, and precision in hand movements (Libertus & Hauf, 2017; Thelen, 2000). Gross, and particularly, fine motor control is closely tied to cognitive development in childhood and predicts outcomes such as executive function, sustained attention, and academic achievement in a variety of domains (Bowler et al., 2024; Cameron et al., 2016; Dinehart & Manfra, 2013; Grissmer et al., 2010; Klupp et al., 2021; Martzog et al., 2019; Piek et al., 2008). Moreover, children with ADHD most commonly present difficulties with fine motor control (Brossard-Racine et al., 2012; Fenollar-Cortés et al., 2017; Goulardins et al., 2015; Klupp et al., 2021; Lin et al., 2024; Rosa Neto et al., 2015; Scott et al., 2024; Tseng et al., 2004). Fine motor control critically enables children to engage in more complex object exploration and engagement in manual production tasks, such as handwriting, which support daily learning activities and literacy development through action (Berninger et al., 2006; Dinehart, 2014; James, 2017; James & Engelhardt, 2012; Kent et al., 2014; Ray et al., 2021; Vinci-Booher et al., in press). Handwriting demands precise fine motor control, which is continually refined throughout childhood and is highly dependent on visual guidance, particularly during the early stages of learning (Fears & Lockman, 2019). The coupling of vision and action during the handwriting process affects the organization of perceptuo-motor brain areas, which may subsequently support learning in other contexts that utilize similar brain systems (Vinci-Booher et al., 2016, in press). Thus, understanding the process of motor development and its relationship to ADHD seems crucial given the importance of action control and cognitive system development in supporting learning and academic achievement. Indeed, academic underachievement is one of the most common and impairing consequences of ADHD, often emerging early in development and persisting across the lifespan (DuPaul & Volpe, 2009). Given that many foundational academic tasks require fine motor control and sensorimotor integration, such as those that require any sort of transcription, as well as the close relationship between fine motor skills in academic achievement, disruptions in fine motor development may be a key, yet under-recognized, contributor to the learning difficulties observed in this population (Bowler et al., 2024; Cameron et al., 2016; Fenollar-Cortés et al., 2017; Grissmer et al., 2010).

## 3. Relationship Between Motor Development and ADHD

Given the functional significance of motor development for cognitive and attentional systems, it is crucial to examine how motor difficulties manifest in children with ADHD. A growing body of research has shown that motor difficulties are prevalent in ADHD, even without the presence of co-morbid DCD and autism, and often emerge early in life (Begum Ali et al., 2020; Landgren et al., 2022; Lee et al., 2021; Lin et al., 2024; Pant et al., 2022). Therefore, these motor difficulties may be more than just co-occurring challenges; rather, they may serve as early markers of ADHD and provide insight into the underlying neurodevelopmental trajectory associated with ADHD. The high rate of co-occurrence between ADHD and DCD further raises the possibility of some shared developmental pathways, particularly in systems supporting motor coordination, attention, and executive function (Wilson et al., 2020).

In the following section, we review evidence on motor impairments in ADHD across early development, focusing on their prevalence, early identification, and potential role in predicting ADHD diagnoses. We concentrate primarily on studies examining motor skill performance and the achievement of motor milestones, as well as sensorimotor integration in infants and children with ADHD. Although less available, we also draw on findings from studies that include children with co-occurring DCD, in order to better contextualize motor difficulties within the broader neurodevelopmental landscape.

### 3.1. Early Motor Development (Table 1)

Research focusing on infant motor behavior and future risk for ADHD is sparse, corresponding with the general lack of attention on motor behavior in ADHD. However, some evidence suggests that increased activity levels, delays in gross motor milestones, and abnormal spontaneous motility may be related to later ADHD diagnosis in early childhood (Athanasiadou et al., 2020; M. H. Johnson et al., 2015; Pant et al., 2022; Reetzke et al., 2022). During infancy, spontaneous motility or generalized, reflexive movements (GMs) of the arms, head, trunk, and legs are present until about 5 months of age on average, and then movements become more controlled and goal directed (Prechtl, 1990). Normally, these movements are complex, vary in speed and intensity, and are “smooth” or fluid. Some evidence suggests that 3–4-month-old infants who exhibited more abnormal GMs, including movements that are invariable, more rigid and jerky, and less fluid overall, were more likely to exhibit symptoms of ADHD later in childhood (Hadders-Algra & Groothuis, 1999). Reetzke et al. (2022) used accelerometry to measure infant locomotor activity levels longitudinally from 12 months to 36 months of age, finding that higher mean levels of limb activity in a variety of naturalistic contexts were related to higher risk of ADHD symptoms at 36 months. On the other hand, a longitudinal study utilizing video recordings found no overall relationship between activity level at 12 months of age and later ADHD diagnosis at 7 years old (P. Johnson et al., 2014). A recent meta-analysis indicated that higher activity levels during infancy were significantly related to ADHD symptoms in childhood, but with high heterogeneity, pointing to issues with inconsistent measurement of motor behavior across studies (Shephard et al., 2022). Limited work has focused on more specific components of action control during infancy; however, some work has measured motor suppression while infants were attending to an object (Friedman et al., 2005; Lawson & Ruff, 2004b). One longitudinal study found that 3-month-olds who exhibited less suppression of body movement upon object gaze onset to an object, followed by a larger rebound of movement after suppression, exhibited higher inattentive symptoms of ADHD at 8 years old compared to typically developing children (Friedman et al., 2005). Overall, these data preliminarily suggest that higher locomotor and gross motor activity are potentially related to later ADHD diagnosis in childhood.

There is mixed evidence of general motor delay in infants who later develop ADHD (Pant et al., 2022; Shephard et al., 2022). Infants at 3 months, 9 months, and 12 months who later developed ADHD in childhood were more likely to exhibit gross motor delays than neurotypical children (Gurevitz et al., 2014; Lemcke et al., 2016). These delays included not maintaining a supine position, exhibiting less head and neck control, and being unable to sit up by 6 months of age (Gurevitz et al., 2014; Lemcke et al., 2016). A recent longitudinal, community-based cohort study found that motor development problems at 10 months, which may include difficulty rolling over, sitting upright independently, putting things into the mouth, and/or crawling forwards or backward, had an increased risk of being diagnosed with ADHD before their 8th birthday (Pant et al., 2022). Some studies suggest that infants at higher risk for ADHD may display either delayed or accelerated motor milestone achievement rather than a consistent pattern of delay (Athanasiadou et al., 2020; Gurevitz et al., 2014; M. H. Johnson et al., 2015). This variability may reflect equifinality, where different developmental pathways lead to similar outcomes, or could indicate the presence of unmeasured comorbidities such as DCD. Despite this heterogeneity, evidence generally supports a link between atypical motor milestone timing in infancy and later ADHD diagnoses. However, other research has shown that motor difficulties can also emerge later in childhood, even in the absence of identifiable motor delays in infancy (Farran et al., 2020), potentially suggesting that motor impairments in ADHD exhibit non-linear or complex origins.

Finally, minimal research has assessed the development of manual dexterity during infancy in the context of ADHD; however, recent work suggests that 10-month-old infants at high genetic risk for ADHD (or have one or more older siblings with ADHD) are less likely to produce manual behaviors that cross the midline compared to low-risk infants (have no older siblings with ADHD) (Begum Ali et al., 2020). Goal-directed manual reaching supports infants’ haptic and visual exploration of their environments, and thus, crossing the midline further increases abilities and opportunities for environmental interactions and supports cognitive development (Adolph & Hoch, 2019; Melzer et al., 2012). In another vein, infants who develop ADHD later in childhood exhibited significantly more speech delays than infants who did not develop ADHD, representing difficulties in oral–motor skills (Gurevitz et al., 2014; Shephard et al., 2022). Overall, action difficulties during infancy seem to be related to an increased likelihood of developing ADHD in children and should be investigated further.

**Table 1:** Key characteristics of early motor development studies in ADHD.

Author(s)	Design Type	Groups & Ages	Motor Measures	Key Findings
(Begum Ali et al., 2020)	Longitudinal	TD infants (*n* = 26) and infants at elevated likelihood of ADHD (*n* = 16), ASD (*n* = 52), ADHD + ASD (*n* = 13); ages 5, 10, and 14 months	Spontaneous midline crossing during a naturalistic reaching task	Infants with higher likelihood of ADHD produced fewer manual behaviors crossing the midline at 10 months old
(Friedman et al., 2005)	Longitudinal	TD infants (*n* = 26) with measures of ADHD symptoms at 8 years old; ages 1 or 3 months old, followed at 8 years old	Body movement during free looking task at 1 or 3 months old	Attention problems at 8 years old associated with less movement suppression upon looking during infancy
(Gurevitz et al., 2014)	Longitudinal	Children diagnosed with (*n* = 58) and without ADHD (*n* = 58); ages 0–1, 3, 9, and 18 months old, followed at school-age	Parent-report fine and gross motor developmental milestones	Gross motor delays at 3 and 18 months associated with later ADHD diagnosis
(Hadders-Algra & Groothuis, 1999)	Longitudinal	Mixed group of infants at low or high risk for neurodevelopmental disorders (*n* = 52); early infancy with follow-up at 4–9 years old	Spontaneous motility in supine position during infancy	Fidgety and mildly abnormal general movements during infancy predicted ADHD in childhood
(P. Johnson et al., 2014)	Longitudinal	Children with (*n* = 16) and without ADHD (*n* = 120); 12-month-old infants with follow-up at 7 years old	Gross motor activity at 12 months old	Higher infant motor activity was not related to ADHD in childhood but was related to inattentive symptoms in boys
(Lawson & Ruff, 2004b)	Longitudinal	Infants with low birth weight (*n* = 55); ages 7 months, then 2, 3, and 4–5 years old	Multimodal object attention during free-play at 7 months old	Worse focused object attention at 7 months old was related to worse cognitive abilities in toddlerhood and higher ADHD symptoms by 4–5 years old
(Lemcke et al., 2016)	Longitudinal	Children diagnosed with ADHD in Danish National Birth Cohort (*n* = 2034); ages 6 and 18 months old, followed at 8–14 years old	Parent-report fine and gross motor developmental milestones	Difficulties with postural stability at 6 months and fine and gross motor difficulties at 18 months related to higher childhood ADHD symptoms
(Pant et al., 2022)	Longitudinal	Children in Child Health Database and Danish National Patient Register Cohort (*n* = 33,238) with multiple diagnoses evaluated; ages 8–10 months old, followed between 1 month and 8 years old	Motor development problems in infancy assessed by nurse at multiple timepoints	Motor development problems during infancy predicted likelihood of being diagnosed with ADHD in childhood
(Reetzke et al., 2022)	Longitudinal	Infants at high and low risk for ADHD and ASD, analyzed into outcome groups of TD (*n* = 77), ASD (*n* = 19), and ADHD (*n* = 17) at 36 months; ages 12, 18, 24, and 36 months	Motor activity measures in structured vs. unstructured play contexts	ADHD and ASD groups exhibited higher motor activity than TD infants at 18 months old

### 3.2. Motor Skills in Childhood (Table 2)

It is estimated that at least half of all children diagnosed with ADHD exhibit significantly more motor difficulties than age-matched, neurotypical peers in a variety of assessments and tasks, with some evidence suggesting a dramatic two-year lag in motor skill development, irrespective of DCD (Farran et al., 2020; Goulardins et al., 2024; Kaiser et al., 2015; Rosa Neto et al., 2015). Interestingly, a recent population-based study suggested that deficits in motor skills and ADHD symptoms during childhood were related to worse functional outcomes at age 30, emphasizing the importance of motor abilities and cascading functional impairment in ADHD throughout the lifespan (Landgren et al., 2022). Regardless, it is important to note that children with DCD and ADHD exhibit worse performance in a variety of gross and fine motor tasks compared to DCD or ADHD alone, although both single diagnoses exhibit difficulties (Pranjić et al., 2023).

Children with ADHD may exhibit worse gross motor skills than typically developing children, including difficulties with balance, catching, kicking and throwing balls, organizing and sequencing gross motor movements in complex tasks, and overall strength and agility (Bünger et al., 2021; D’Anna et al., 2024; Pitcher et al., 2003; Rosa Neto et al., 2015; Tseng et al., 2004). Poor balance and postural control are the most consistently reported gross motor issues in children with ADHD, with deficits present during a variety of simple and complex locomotor tasks (e.g., walking backward and hopping and alternating between each foot) (Buderath et al., 2009; Bünger et al., 2021; Kroes et al., 2002; Tseng et al., 2004). Worse gross motor skills, especially locomotor difficulties, predicted a higher risk of ADHD in school-aged children, with worse locomotor skills relating to higher inattentive symptoms, and ball skill difficulty relating to more hyperactive/impulsive symptoms (D’Anna et al., 2024). Higher mean gross motor activity and less variation in movement as measured by accelerometry were related to higher ADHD symptoms and more impairment in classroom contexts (Shoulberg et al., 2024). Some limited research suggests that sensorimotor integration difficulties may underlie poorer locomotor and balance performance in children with ADHD (Shum & Pang, 2009). Thus, there is compelling evidence that children with ADHD seem to have a higher risk of gross motor difficulties, which may be compounded by early gross motor skill development.

Fine motor skills and manual dexterity seem to be the most challenging for children with ADHD, with some evidence pointing to worse abilities in those with higher inattentive symptoms (Brossard-Racine et al., 2012; Fenollar-Cortés et al., 2017; Goulardins et al., 2015; Klupp et al., 2021; Lin et al., 2024; Rosa Neto et al., 2015; Scott et al., 2024; Tseng et al., 2004). Children with ADHD exhibit worse accuracy, fluency, and speed in manual and fine motor dexterity tasks, such as the grooved pegboard, pen and paper tracing, finger succession, and knot tying, and exhibit more subtle motor signs compared to typically developing children (Akkaya et al., 2025; Bünger et al., 2021; Carames et al., 2022; Farran et al., 2020; Fenollar-Cortés et al., 2017; Goulardins et al., 2017; Hyde et al., 2024; Hyde et al., 2021b; Lin et al., 2024; Mokobane et al., 2019; Rosa Neto et al., 2015; Scott et al., 2024; Tseng et al., 2004). Interestingly, these difficulties often remain even after stimulant medication treatment, as well as while controlling for age and sex, indicating that this is not merely a developmental delay (Akkaya et al., 2025; Kaiser et al., 2015; Klupp et al., 2021).

Early fine motor and manual skills enable academic achievement in children, which is important to consider as children with ADHD frequently present with academic difficulties (Cameron et al., 2016; DuPaul & Volpe, 2009; Grissmer et al., 2010). One recent study found that worse fine motor skills in preschool-aged children predicted higher ADHD traits and genetic risk as well as lower educational achievement in late childhood and adolescence (Bowler et al., 2024). Additionally, ADHD risk status, as well as fine and gross motor skills, uniquely predicted school functioning in a sample of school-aged children (Scott et al., 2024). Children with ADHD also exhibit significant difficulties in specific manual skills important for academic achievement, particularly handwriting (Borella et al., 2011; Brossard-Racine et al., 2015; Duda et al., 2019; Farhangnia et al., 2020; Fenollar-Cortés et al., 2017). Children with ADHD often have difficulties with handwriting, including worse accuracy and illegibility, slower speed, and more inconsistent movements, and have general difficulties in written language compared to typically developing peers (Borella et al., 2011; Brossard-Racine et al., 2015; Carames et al., 2022; Duda et al., 2019; Farhangnia et al., 2020; Fenollar-Cortés et al., 2017). Overall, there is compelling evidence suggesting that fine and manual dexterity problems are prevalent in children with ADHD.

Difficulties in visuomotor integration in the context of fine motor tasks are also common in ADHD and have been proposed to drive differences in motor skill performance (Brossard-Racine et al., 2012). Tasks such as drawing or handwriting require ongoing sensorimotor integration to guide movements in real time and adjust based on perceptual feedback. There is some evidence pointing to impaired sensorimotor integration in children with ADHD, in addition to fine motor difficulties (Brossard-Racine et al., 2012; Carames et al., 2022; Egeland et al., 2012; Farhangnia et al., 2020; Kaiser et al., 2015). Moreover, many of the tasks used to assess fine and manual dexterity in ADHD rely on visuomotor integration, even if not explicitly framed as such. This is important to consider as disruptions in these processes may impair not only motor skill acquisition but also the development of fluency and automaticity that comes from repeated sensorimotor coordination. Previously, sensorimotor integration has been deemed a distinct mechanism related to DCD, not ADHD-related motor difficulties (Piek & Dyck, 2004). However, the emerging evidence related to visuomotor integration problems in ADHD, particularly in tasks requiring fine motor control, raises the possibility of some shared or interacting mechanisms among the two conditions, although more research examining co-occurring DCD and ADHD is needed (Pranjić et al., 2023).

**Table 2:** Key characteristics of motor behavior studies in children with ADHD.

Author(s)	Design Type	Groups & Ages	Motor Measures	Key Findings
(D’Anna et al., 2024)	Cross-Sectional	Children in primary school sample with varying ADHD symptoms (*n* = 2677); ages 5 to 7 years old	Gross motor development scale, evaluating locomotor and ball skills	Worse motor skills were related to higher symptoms of ADHD and higher risk of ADHD diagnosis
(Farran et al., 2020)	Cross-Sectional	Children with (*n* = 43) and without ADHD (*n* = 34); ages 8 to 15 years old	Measures of fine, gross, and postural stability using standardized tests and motor milestone achievement	45% of children with ADHD exhibited motor impairment, but it was not related to ADHD symptoms specifically. No evidence of motor delay in infancy
(Goulardins et al., 2024)	Cross-Sectional	Children with ADHD (*n* = 14) and ADHD + DCD (*n* = 13); ages 7 to 9 years old	Motor assessment battery and motor development milestone scales	Children with DCD+ADHD exhibited the worst motor performance in fine and balance tasks, both groups had pronounced motor delays
(Kroes et al., 2002)	Longitudinal	Community sample of children (*N* = 401); ages 5 to 6 years old at baseline	Motor assessment focused on balance, ball skills, manual dexterity	Balance and fine motor dexterity predicted ADHD symptoms 18 months after baseline
(Landgren et al., 2022)	Longitudinal	A community sample enriched with children with DCD/motor difficulties + ADHD (*n* = 62) and matched NT controls (*n* = 51); age 9 years old at baseline	Global motor scores from physicians, parents, children, and teachers	Worse neuromotor functioning at 9 years old, in addition to ADHD, predicted significant variance in adult adverse outcomes
(Lin et al., 2024)	Cross-Sectional	Community sample (*N* = 1897) with children at risk for a variety of developmental delays, including ADHD (*n* = 234), DCD (*n* = 128), and TD (*n* = 52); ages 3 to 6 years old	Movement assessment scales that measure fine motor skills	Children with ADHD exhibited poorer fine motor performance compared to TD children, but better performance than other children with developmental delays, such as DCD
(Mokobane et al., 2019)	Cross-Sectional	Children with (*n* = 160) and without (*n* = 160) ADHD; ages 8 to 12 years old	Grooved pegboard and maze coordination tasks to assess fine motor dexterity	Children with higher ADHD symptoms exhibited worse performance on grooved pegboard than TD controls, particularly combined and inattentive symptoms presenting
(Pitcher et al., 2003)	Cross-Sectional	Boys with (*n* = 104) and without ADHD (*n* = 39), ADHD + DCD (*n* = 55); ages 7 to 12 years old	Movement Assessment Battery for Children, grooved pegboard	Children with ADHD performed worse than TD controls in assessments examining movement ability, and ADHD + DCD exhibited worse fine motor skills
(Scott et al., 2024)	Cross-Sectional	Elementary school children (*N* = 202) with 46.5% deemed at-risk for ADHD; ages 4 to 8 years old	Bruininks–Oseretsky Test of Motor Proficiency to measure fine and gross motor skills	Worse fine motor ability was related to higher risk of ADHD, as well as worse academic achievement
(Shoulberg et al., 2024)	Cross-Sectional	Preschoolers with various ADHD symptoms (*N* = 141); ages 3 to 6 years old	Motor activity via accelerometry at school	Higher levels and less variation of motor activity were related to ADHD hyperactive/impulsive symptoms
(Shum & Pang, 2009)	Cross-Sectional	Children with (*n* = 43) and without (*n* = 50) ADHD; ages 6 to 12 years old	Sensory organization of standing balance was evaluated	Children with ADHD exhibited worse postural stability, driven by disruption of sensory signals
(Tseng et al., 2004)	Cross-Sectional	Children with (*n* = 42) and without ADHD (*n* = 42); ages 6 to 11 years old	Bruininks–Oseretsky Test of Motor Proficiency to assess fine and gross motor skills; parent-report activity	Children with ADHD exhibited worse fine and gross motor skills than controls
(Akkaya et al., 2025)	Cross-Sectional	Children with (*n* = 146) and without (*n* = 213) ADHD; ages 7 to 17 years old	Functional dexterity test to assess hand skills	Children with ADHD exhibited worse fine motor dexterity compared to controls
(Bowler et al., 2024)	Longitudinal	Preschool children from Twins Early Development Study (*N* = 9625); ages 2, 3, 4; 7–8, 12, and 16 years old	Fine motor assessments of drawing, block building, folding, and questionnaires were assessed at younger ages, neurodevelopmental traits were assessed later	Lower fine motor skills in early childhood predicted higher risk for ADHD later in childhood, as well as educational achievement
(Bünger et al., 2021)	Cross-Sectional	Children with (*n* = 52) and without (*n* = 52) ADHD; ages 6 to 13 years old	Movement Assessment Battery for Children assessing fine and gross motor skills, as well as DCD symptoms	Children with ADHD exhibited worse fine and gross motor skills and higher levels of DCD symptoms compared to controls
(Carames et al., 2022)	Cross-Sectional	Children with (*n* = 28) and without (*n* = 11) ADHD; ages 8 to 13 years old	Assessment of visuomotor integration	Children with ADHD exhibited lower visuomotor integration and fine motor control, but not visual perception
(Egeland et al., 2012)	Cross-Sectional	Children with (*n* = 67) and without (*n* = 67) participated; ages 9 to 16 years old	Visuomotor integration and grooved pegboard/finger tapping for manual dexterity	Children with ADHD exhibited deficits in visuomotor integration and manual dexterity relative to controls
(Fenollar-Cortés et al., 2017)	Cross-Sectional	Children with (*n* = 43) and without (*n* = 42) ADHD; ages 7 to 14 years old	A variety of standardized tasks assessing fine motor control/dexterity	Children with ADHD performed worse across all fine motor tasks compared to controls, particularly those with higher inattentive symptoms
(Klupp et al., 2021)	Cross-Sectional	Children with (*n* = 46) and without ADHD (*n* = 139); ages 7 to 13 years old	Movement Assessment Battery for Children to assess fine motor control	Children with ADHD exhibited worse fine motor skills compared to controls
(Rosa Neto et al., 2015)	Cross-Sectional	Children with (*n* = 50) and without (*n* = 150) ADHD; ages 5 to 10 years old	Standardized assessment for motor development	Children with ADHD exhibit significant delay in motor development compared to controls, particularly in fine motor skills

### 3.3. Neuroimaging Evidence in Childhood (Table 3)

Limited research utilizing neuroimaging provides converging evidence that children with ADHD exhibit functional and structural differences related to a distributed neural system supporting motor control (e.g., basal ganglia, cerebellum, and frontal and parietal cortices) when compared to neurotypical children. Children with ADHD and/or DCD exhibited lower functional connectivity (FC) between the primary motor cortex and angular gyri within the frontoparietal network, as well as reduced FC between the primary motor cortex and the basal ganglia, perhaps indicating shared neural substrates for both motor and attention problems (McLeod et al., 2014). Another study examining neural substrates among children with ADHD and/or DCD indicated that these groups all exhibited lower activation in the right primary motor, right sensory cortices, and left superior and middle frontal gyri compared to neurotypical controls during a motor response inhibition task (Thornton et al., 2018). Interestingly, children with DCD and/or ADHD all exhibited poorer cognitive task performance compared to controls (Thornton et al., 2018). Children with ADHD exhibited lower activation of the frontoparietal network and motor cortex activation during finger tapping and motor inhibition tasks compared to neurotypical children (with no differences in performance) (Dickstein et al., 2006; Mostofsky et al., 2006).

Children with ADHD seem to exhibit structural and functional differences in the cerebellum. Metanalyses indicate that children with ADHD exhibit lower cerebellar volume and lower FC within the fronto-striatal-cerebellar pathway compared to neurotypical children (Cortese & Castellanos, 2012; Cortese et al., 2012; Dickstein et al., 2006; Valera et al., 2007). Additionally, children with ADHD compared to neurotypical controls exhibit increased cerebro-cerebellar FC as a function of age and overall showed higher cerebro-cerebellar FC in the superior temporal gyrus within the somatomotor network (Wang et al., 2022). Another study showed that children with ADHD exhibit less white matter integrity in the right cerebral peduncle, left middle cerebellar peduncle, and left cerebellum (Ashtari et al., 2005), suggesting disrupted structural connectivity that may contribute to motor and cognitive difficulties.

Emerging work suggests that white matter organization and development reflect differential symptomatic and motor outcomes for children with ADHD, perhaps providing early evidence that motor difficulties may be a core feature rather than a secondary effect. Atypical microstructure organization of the corticospinal tract (CST), a key white matter pathway supporting motor control, is associated with higher ADHD symptoms in children, as well in children with ADHD and/or DCD-related motor difficulties, and adolescents with ADHD with persistent motor difficulties (Hyde et al., 2021a, 2023). Indeed, poorer CST integrity in children with ADHD is rescued by adolescence with improved motor dexterity skills (Hyde et al., 2023). These findings underscore the dynamic and experience-dependent nature of white matter development: CST maturation has been shown to be driven in part by increased skill and engagement with fine motor activities, independent of chronological age (Fuelscher et al., 2021). Such evidence supports a model in which motor system development scaffolds broader cognitive and behavioral functions central to ADHD. For example, children with ADHD exhibited worse fine motor dexterity compared to neurotypical controls, which was related to lower white matter density and volume in the superior longitudinal fasciculus (SLF), a fiber tract connecting frontal, parietal, and occipital cortices, and has been implicated in motor and attentional control (Hyde et al., 2021a, 2021b). Other longitudinal work has indicated that CST integrity predicts symptom improvement throughout childhood into adolescence, with ADHD symptom remission relating to accelerated fiber development in the CST, fronto-pontine, striatal-premotor, and thalamo-premotor tracts, while persistent ADHD into adolescence showed ongoing alterations in these sensorimotor pathways (Damatac et al., 2022; Francx et al., 2015; Fuelscher et al., 2023; Leenders et al., 2021). Together, these findings preliminarily suggest that the integrity and development of motor-related white matter pathways may serve as a neurobiological foundation upon which attentional and executive functions are built.

**Table 3:** Key characteristics of motor-related neuroimaging studies in children with ADHD.

Author(s)	Design Type	Groups & Ages	Method	Key Findings
(Ashtari et al., 2005)	Cross-Sectional	Children with (*n* = 18) and without (*n* = 15) ADHD; ages 7 to 10 years old	Resting State; DTI	Children with ADHD exhibited reduced white matter integrity in key cerebellar pathways compared to controls
(Damatac et al., 2022; Francx et al., 2015; Leenders et al., 2021)	Longitudinal	Children with, at-risk, or without ADHD; ages 6 to 18, 9 to 26; 12 to 29; 18 to 34	Resting State; DTI	ADHD symptom remission was associated with accelerated fiber development in sensorimotor tracts into adolescence and adulthood; persistent ADHD showed ongoing alterations in these pathways
(Wang et al., 2022)	Cross-Sectional	Children and adolescents with (*n* = 106) and without (*n* = 62) ADHD; ages 8 to 16	Resting State FC	ADHD group showed higher FC in superior temporal gyrus and increasing cerebro-cerebellar FC with age
(Mostofsky et al., 2006)	Longitudinal	Children with (*n* = 11); and without (*n* = 11) ADHD; ages 8 to 12	Task-Based fMRI with finger tapping	ADHD group showed decreased contralateral motor cortex and right parietal cortex activation during task
(McLeod et al., 2014)	Cross-Sectional	Children with ADHD (*n* = 21), DCD (*n* = 7), DCD + ADHD (*n* = 18) and controls (*n* = 23); ages 8 to 17	Resting State FC	Children with ADHD, DCD, and DCD+ADHD exhibited lower FC in motor networks compared to controls
(Thornton et al., 2018)	Cross-Sectional	Children with ADHD (*n* = 20), DCD (*n* = 9), DCD + ADHD (*n* = 18) and controls (*n* = 20); ages 8 to 17	Task-Based fMRI with go–no-go task	Lower activation in right primary motor, right sensory cortex, and left frontal gyri; all clinical groups showed worse cognitive task performance
(Fuelscher et al., 2023)	Longitudinal	Children with persistent ADHD (*n* = 62), remitted ADHD (*n* = 37), and controls (*n* = 85); ages 10 at first wave, and ~18 months for 3 waves	Resting State; DTI	Persistent ADHD later in showed ongoing white matter alterations along sensorimotor pathway compared to remitted and control groups
(Hyde et al., 2021a)	Cross-Sectional	Children with (*n* = 50) and without (*n* = 56) ADHD; ages 9–11	Resting State; DTI with grooved pegboard	Children with ADHD exhibited worse fine motor dexterity than controls, and also exhibited lower CST integrity
(Hyde et al., 2021b)	Cross-Sectional	Children with (*n* = 55) and without (*n* = 61) ADHD; ages 9–11	Resting State; DTI with grooved pegboard	Children with ADHD exhibited worse fine motor dexterity with dominant hand, which corresponded to lower SLF integrity
(Hyde et al., 2023)	Longitudinal	Children with (*n* = 27) and without (*n* = 33) ADHD; ages 9–14 with 3 waves of imaging	Resting State; DTI	In middle childhood, children with ADHD exhibited lower BL CST integrity relative to those without ADHD, whether they had motor difficulties or not; and CST integrity improved in children with ADHD without motor difficulties in adolescence
(Hyde et al., 2024)	Cross-Sectional	Children with (*n* = 92) and without (*n* = 185) ADHD; ages 8–12	Resting State; DTI; measured subtle motor signs	Morphology of sensorimotor tracts contributes to severity of subtle motor signs in children with and without ADHD, but not unique to ADHD

### 3.4. Motor-Based Interventions and ADHD

Research on motor interventions provides valuable insight into the relationship between the motor system and ADHD by highlighting how targeted training can influence both motor and cognitive outcomes. Recent work suggests that motor-based interventions, including fine motor interventions (e.g., handwriting training and visuomotor skills) and gross motor interventions (e.g., structured vs. unstructured exercise training) are generally efficacious for children with ADHD in improving targeted motor skills (Kleeren et al., 2023; Lelong et al., 2021). Interestingly, fine motor interventions have also been shown to improve core ADHD symptoms and executive functioning, above and beyond medication use (Lelong et al., 2021), and gross motor interventions also facilitate improvements in executive functioning in children with ADHD (Qiu et al., 2024). Thus, improvements in motor skills and subsequent symptomatic and cognitive improvement in children with ADHD suggest an intertwined relationship between action and cognition in this population.

## 4. Applying Embodied Frameworks

Despite years of accumulated research suggesting an inseparable nature between perception and action, two predominant theories within the ADHD literature continue to offer overly simplistic explanations for why motor difficulties are common in children with ADHD. The first theory suggests that motor problems are unidirectionally caused by the core symptoms of ADHD, particularly difficulties in executive functioning (Damatac et al., 2022; Goulardins et al., 2017; Kaiser et al., 2015; Lelong et al., 2021; Willcutt et al., 2005, 2012). Indeed, motor learning and performance require executive functions, such as inhibitory control, to coordinate and monitor movements, extract sensory information from the environment, and generally sustain effort on a given task (Song, 2019). Within this framework, delays in executive functioning are presumed to impair motor learning and performance in a top-down fashion. Supporting this view, many purely cognitive models of ADHD generally emphasize that difficulty with executive functioning is the core mechanism underlying ADHD symptomology, attributing these deficits to dysregulated dopamine transmission resulting from complex gene–environment interactions (del Campo et al., 2011; Fusar-Poli et al., 2012; Wu et al., 2012).

The second prevalent theory similarly treats motor difficulties in ADHD as secondary–this time attributing them to co-occurring DCD, a condition formally considered a motor disorder rather than a “cognitive” disorder, like ADHD (Goulardins et al., 2017; Pant et al., 2022). From this perspective, motor difficulties are not an important feature contributing to an ADHD phenotype, but rather are the result of comorbid DCD, which reflects delayed or atypical maturation of the motor system. Supporting this, some researchers argue that motor difficulties are too heterogenous or subtle to be considered a core feature of ADHD (Athanasiadou et al., 2020; Goulardins et al., 2013, 2017, 2015). Others have argued that the inconsistent inclusion of DCD symptoms in ADHD motor research has muddled our understanding of whether motor impairments are truly a part of ADHD or merely a result of overlapping symptoms (Meachon et al., 2025).

However, a small but growing body of evidence shows that even when DCD symptoms are controlled for, children with ADHD still demonstrate meaningful motor difficulties, though these may be less severe than in children with comorbid DCD or DCD alone (Brossard-Racine et al., 2012; Goulardins et al., 2017, 2024; McLeod et al., 2014; Pranjić et al., 2023). Yet, high rates of comorbidity are often interpreted as evidence that motor difficulties in ADHD are either indistinguishable from or entirely secondary to DCD, rather than prompting inquiry into shared, distinct, or interactive developmental mechanisms. Such interpretations risk oversimplifying the developmental origins of motor difficulties in ADHD by assuming a clear boundary between cognitive and motor domains. In doing so, they neglect the possibility that ADHD-related motor challenges may emerge from unique interactions between perception, action, and cognitive systems, not simply from a delay in motor system maturation (Karmiloff-Smith, 2013).

It is also important to acknowledge that not all children with ADHD exhibit overt or clinically significant motor difficulties (Athanasiadou et al., 2020; Goulardins et al., 2013). However, this does not mean that sensorimotor skills are unaffected in these children or were not important in shaping their individual developmental trajectory. Even in the absence of overt motor delays, some children may experience subtle disruptions in their sensorimotor interactions that may impact their cognitive and behavioral development, perhaps in specific environments with more complex demands. The relationship between motor and cognitive difficulties in ADHD is dynamic and complex, which would lead to variability in how ADHD symptoms may manifest in early childhood (Byrge et al., 2014). Indeed, different developmental pathways may lead to similar outcomes, such as attentional and behavioral difficulties, despite differences in the presence or severity of motor impairments. Importantly, many standardized assessments emphasize performance delays or errors rather than capturing how motor and sensorimotor behaviors function in real-time interaction with the environment (Kretch et al., 2024). Furthermore, protective factors, such as early interventions or system inputs in the developmental cascade, may prevent the development of overt motor difficulties, even when subtle disruptions to sensorimotor skills exist (N. A. Koziol et al., 2023). Thus, we should investigate sensorimotor experiences as continuous, complex, developmentally significant processes that shape cognitive and behavioral trajectories, rather than relying solely on simple, categorical definitions of motor disorder.

Ultimately, both of these dominant theories take a reductionistic, linear view of motor difficulties in ADHD and generally disregard the tight coupling between motor, cognitive, and behavioral development, as previously described. The executive functioning theory implies a top-down, causal relationship by which cognitive deficits precede and explain motor impairments, failing to account for the bidirectional and reciprocal nature of development (Karmiloff-Smith, 2013). It also contradicts neurodevelopmental evidence indicating that motor systems emerge earlier and critically scaffold the development of higher-order cognitive process development (Gottwald et al., 2016; M. H. Johnson & de Haan, 2015; Rosen et al., 2019). Similarly, the DCD theory is too simple in that in order to consider motor problems in ADHD to be truly “motor” in nature, they must reflect DCD pathology, thereby separating motor from cognitive difficulties as distinct and unrelated, despite evidence indicating that many children with ADHD and without DCD exhibit difficulties in motor control.

Both perspectives take a perhaps over-simplified, linear approach to understanding the motor difficulties in ADHD, and critically, do not describe the mechanisms by which these motor difficulties emerge, as well as co-morbidity with DCD. Specifically, they ignore developmental research showing that motor development and sensorimotor experiences—movement, object interaction, and perceptually guided action—actively shape neural and cognitive development across early childhood (Byrge et al., 2014; Kaur et al., 2022; Thelen, 2000). These embodied experiences are not merely downstream outputs of cognition or just motor developmental milestones, but rather, they are formative inputs that structure cognitive systems themselves. Thus, a more comprehensive framework is needed to help disentangle the complex phenomenon of ADHD—one that accounts for the dynamic, bidirectional relationships between motor behavior, cognitive development, and environmental interaction.

Embodied cognition offers such a framework. It moves beyond traditional dichotomies between “motor” and “cognitive” processes, and instead, proposes that bodies and perceptually guided actions are fundamentally intertwined with cognitive and neural systems and that their development is mutually constitutive. This approach situates motor development not as a consequence of internal cognitive delays or isolated motor deficits, but rather as a core mechanism by which cognitive systems, such as attention and executive function, are built and refined. By embedding the child within a context of dynamic, sensorimotor interactions within an environment, this framework may help explain the emergence of motor and cognitive challenges present in ADHD as interdependent outcomes of a disrupted developmental cascade (Karmiloff-Smith, 2013). As such, an embodied theoretical framework is uniquely positioned to help illuminate how differences in motor difficulties give rise to attentional, academic, and behavioral difficulties that characterize ADHD across the lifespan.

## 5. Conclusions and Future Directions

The process of development requires a broader, temporal consideration of an extended brain–body–behavior system (Byrge et al., 2014). Given that ADHD is a neurodevelopmental disorder, it is essential to apply the theoretical lens that more embodied, dynamical systems theories offer. From this perspective, motor difficulties in ADHD are not isolated impairments or mere byproducts of core attentional or executive deficits, but rather they reflect disruptions in action systems that fundamentally shape how children engage with, learn from, and adapt to their environments. Specifically, we propose that perhaps motor difficulties and cognitive challenges in ADHD may co-emerge from the same underlying disruptions in sensorimotor processes and environmental exploration, rather than existing in a unidirectional or secondary relationship. Potentially, these disruptions can be identified at key developmental transition points or sensitive periods during infancy or early childhood (e.g., the onset of sitting, reaching, or fine motor coordination) before the core symptoms of ADHD fully manifest.

In order to empirically test this hypothesis, longitudinal studies are needed that track motor and cognitive development from infancy through early childhood utilizing multiple levels of analysis. Specifically, we hypothesize that (1) children diagnosed later with ADHD will show atypical patterns of motor behaviors or action control (e.g., delayed sitting, less stable postural control, and lower variability in reaching) during infancy and toddlerhood prior to observable attention or executive function difficulties. (2) These differences in behavior may predict later attentional or executive function difficulties, as well as core ADHD symptoms, even while controlling for age and baseline cognitive ability. (3) More variation in action control and environmental exploration early in development will relate to differences in neural activity in sensorimotor and frontoparietal networks.

Moreover, key developmental transitions, such as the onset of independent locomotion or the refinement of manual skills, may represent periods of heightened neural plasticity, and thus, subtle differences in motor behavior produce cascading effects on environmental exploration, learning, and behavioral outcomes, which may be adaptive or maladaptive in the case of ADHD (Byrge et al., 2014; Smith & Thelen, 2003; Van Geert, 2011). Therefore, observations of how skills both emerge and stabilize across these key transition periods may provide insight into whether motor and cognitive difficulties represent parallel adaptations to early sensorimotor difficulties, rather than serial consequences of isolated deficits (Kretch et al., 2024). Importantly, these predictions are distinct from traditional executive function theories, which posit that motor impairments are downstream of frontoparietal dysfunction, and the DCD theory that attributes motor problems exclusively to co-morbidity. Our hypothesis will facilitate the examination of shared vs. distinct developmental trajectories between motor and cognitive difficulties in a dimensional manner, and thus, can help identify early markers that precede categorical diagnosis.

So far, much of the work examining motor development and ADHD has utilized cross-sectional or correlational designs or has been reliant on clinical samples in later childhood. There is also a lack of examination of DCD in the context of ADHD, which is important to understand the potential shared or distinct mechanisms. Thus, to implement an embodied framework, future work should employ rich, longitudinal, and multi-modal designs that examine changes in motor behavior, cognitive ability, and neural responses over time. This would allow researchers to track developmental trajectories, identify key inflection points, and assess the stability or reorganization of action systems across early development. In addition to utilizing multiple levels of analysis that capture change across varying timescales and modalities (e.g., head-mounted eye-tracking, neuroimaging, self-report measures, and accelerometry), we also argue for more research in naturalistic contexts that allow for more ecologically valid and dynamic measures of action control.

A theoretically enriched, embodied framework has powerful implications for how we understand, identify, and intervene in ADHD and other neurodevelopmental disorders. An embodied perspective acknowledges the motor difficulties common in ADHD and repositions them as potential integral components of ADHD’s neurodevelopmental profile, not just co-occurring symptoms. This shift also has profound clinical and public health implications, as it may inspire earlier identification of ADHD based on specific sensorimotor markers, as well as interventions that target sensorimotor coordination at key points in development, which may positively influence cognitive and behavioral outcomes. Importantly, an embodied approach underscores the need for interdisciplinary collaboration across pediatrics, developmental and clinical science, neuroscience, and education.

By moving beyond traditional cognitive models and embracing embodied perspectives, we not only enrich the theoretical model of ADHD but also expand the range of tools available to support children with neurodevelopmental differences. Ultimately, understanding the tight coupling between body, brain, and behavior throughout development will be critical to developing effective interventions that are developmentally timed and mechanistically grounded, ultimately improving the lives and outcomes of individuals with ADHD.

## Abbreviations

The following abbreviations are used in this manuscript:

ADHD	Attention-deficit/hyperactivity disorder
DCD	Developmental coordination disorder
